# Prophylactic radiotherapy for the prevention of procedure-tract metastases after surgical and large-bore pleural procedures in malignant pleural mesothelioma (SMART): a multicentre, open-label, phase 3, randomised controlled trial

**DOI:** 10.1016/S1470-2045(16)30095-X

**Published:** 2016-08

**Authors:** Amelia O Clive, Hazel Taylor, Lee Dobson, Paula Wilson, Emma de Winton, Niki Panakis, Justin Pepperell, Timothy Howell, Samuel A Stewart, Erika Penz, Nikki Jordan, Anna J Morley, Natalie Zahan-Evans, Sarah Smith, Timothy J P Batchelor, Adrian Marchbank, Lesley Bishop, Alina A Ionescu, Mike Bayne, Samantha Cooper, Anthony Kerry, Peter Jenkins, Elizabeth Toy, Vallipuram Vigneswaran, James Gildersleve, Merina Ahmed, Fiona McDonald, Mick Button, Conrad Lewanski, Charles Comins, Muthukumar Dakshinamoorthy, Y C Gary Lee, Najib M Rahman, Nick A Maskell

**Affiliations:** a Respiratory Research Unit, North Bristol National Health Service (NHS) Trust, Southmead Hospital, Bristol, UK; b Academic Respiratory Unit, School of Clinical Sciences, University of Bristol, Bristol, UK; c Research Design Service South West, Bristol, UK; d South Devon Healthcare NHS Foundation Trust, Torbay, UK; e University Hospitals Bristol NHS Trust, Bristol, UK; f Royal United Hospital, Bath, UK; g Oxford University Hospitals NHS Trust, Oxford, UK; h Musgrove Park Hospital, Taunton, UK; i Plymouth Hospitals NHS Trust, Plymouth, UK; j Faculty of Medicine, Dalhousie University, Halifax, NS, Canada; k Department of Medicine, University of Saskatchewan, Saskatoon, SK, Canada; l Portsmouth Hospitals NHS Trust, Portsmouth, UK; m Royal Gwent Hospital, Aneurin Bevan University Health Board, Newport, UK; n Poole Hospital NHS Foundation Trust, Poole, UK; o Dorset County Hospital NHS Foundation Trust, Dorchester, UK; p Colchester Hospital University NHS Foundation Trust, Colchester, UK; q Great Western Hospitals NHS Trust, Swindon, UK; r Gloucestershire Hospitals NHS Foundation Trust, Gloucester, UK; s Royal Devon and Exeter NHS Foundation Trust, Exeter, UK; t Singleton Hospital, Swansea, UK; u Withybush Hospital, Haverfordwest, UK; v Royal Berkshire NHS Foundation Trust, Reading, UK; w The Royal Marsden NHS Foundation Trust, London, UK; x Nevill Hall Hospital, Abergavenny, UK; y Imperial College Healthcare NHS Trust, London, UK; z Centre for Respiratory Health, School of Medicine & Pharmacology, University of Western Australia, Perth, WA, Australia; aa Oxford National Institute for Health Research (NIHR) Biomedical Research Centre, Oxford, UK

## Abstract

**Background:**

The use of prophylactic radiotherapy to prevent procedure-tract metastases (PTMs) in malignant pleural mesothelioma remains controversial, and clinical practice varies worldwide. We aimed to compare prophylactic radiotherapy with deferred radiotherapy (given only when a PTM developed) in a suitably powered trial.

**Methods:**

We did a multicentre, open-label, phase 3, randomised controlled trial in 22 UK hospitals of patients with histocytologically proven mesothelioma who had undergone large-bore pleural interventions in the 35 days prior to recruitment. Eligible patients were randomised (1:1), using a computer-generated sequence, to receive immediate radiotherapy (21 Gy in three fractions within 42 days of the pleural intervention) or deferred radiotherapy (same dose given within 35 days of PTM diagnosis). Randomisation was minimised by histological subtype, surgical versus non-surgical procedure, and pleural procedure (indwelling pleural catheter *vs* other). The primary outcome was the incidence of PTM within 7 cm of the site of pleural intervention within 12 months from randomisation, assessed in the intention-to-treat population. This trial is registered with ISRCTN, number ISRCTN72767336.

**Findings:**

Between Dec 23, 2011, and Aug 4, 2014, we randomised 203 patients to receive immediate radiotherapy (n=102) or deferred radiotherapy (n=101). The patients were well matched at baseline. No significant difference was seen in PTM incidence in the immediate and deferred radiotherapy groups (nine [9%] *vs* 16 [16%]; odds ratio 0·51 [95% CI 0·19–1·32]; p=0·14). The only serious adverse event related to a PTM or radiotherapy was development of a painful PTM within the radiotherapy field that required hospital admission for symptom control in one patient who received immediate radiotherapy. Common adverse events of immediate radiotherapy were skin toxicity (grade 1 in 50 [54%] and grade 2 in four [4%] of 92 patients *vs* grade 1 in three [60%] and grade 2 in two [40%] of five patients in the deferred radiotherapy group who received radiotherapy for a PTM) and tiredness or lethargy (36 [39%] in the immediate radiotherapy group *vs* two [40%] in the deferred radiotherapy group) within 3 months of receiving radiotherapy.

**Interpretation:**

Routine use of prophylactic radiotherapy in all patients with mesothelioma after large-bore thoracic interventions is not justified.

**Funding:**

Research for Patient Benefit Programme from the UK National Institute for Health Research.

## Introduction

Malignant pleural mesothelioma is an aggressive tumour with a poor prognosis, and few treatment options are available. In 2012, 2535 mesothelioma deaths were reported in the UK alone, and the incidence is predicted to increase.^1^, ^2^ Patients often have multiple diagnostic and therapeutic pleural interventions to confirm the diagnosis and manage symptomatic malignant pleural effusions. However, a complication of these procedures is that the tumour can spread to the site of previous interventions, resulting in procedure-tract metastases (PTMs). Data from a retrospective study^3^ suggest that the risk of these potentially painful nodules developing increases with the size of the chest wall incision.

Mesothelioma is sensitive to radiation therapy in vitro,^4^ but its use as a radical treatment is limited by the dose that can be delivered safely to thoracic organs at risk. Prophylactic radiotherapy to pleural intervention sites can be given with minimal side-effects; however, results from three small randomised controlled trials^5^, ^6^, ^7^ assessing its efficacy in reducing PTMs are conflicting and showed substantial variation in PTM incidence. Three meta-analyses^8^, ^9^, ^10^ of these data concluded that there was insufficient evidence to recommend the use of prophylactic radiotherapy. Specifically, there is a paucity of data regarding the incidence of clinically relevant, symptomatic nodules. These factors have resulted in variation in clinical practice^8^, ^11^ worldwide and in international guidelines;^12^, ^13^, ^14^, ^15^, ^16^, ^17^ therefore, a suitably powered randomised controlled trial is needed.

**Research in context**

**Evidence before this study**

We searched MEDLINE, Embase, CINAHL, and AMED for articles published until Sept 16, 2015, that reported prophylactic radiotherapy in mesothelioma. We used the keywords “mesothelioma”, “neoplasms, mesothelial”, and “Prophylactic Radiotherap* OR irradiation OR radiation therapy”. We assessed titles, keywords, and abstracts, and obtained full texts of articles that we deemed relevant. We used the Critical Appraisal Skills Programme checklist to assess the quality of the evidence. We identified three published randomised controlled trials and one additional randomised controlled trial that is still in the follow-up period. We found three systematic reviews incorporating evidence from randomised trials and non-randomised studies; all three reviews concluded that there was insufficient evidence to recommend prophylactic radiotherapy in mesothelioma and called for a large-scale randomised controlled trial to be done.

**Added value of this study**

Our results show that prophylactic radiotherapy to large-bore pleural intervention sites does not confer benefits in terms of the rate of procedure-tract metastasis (PTM), chest pain, quality of life, analgesia use, or survival.

**Implication of all the available evidence**

Taken together with results from the three previous randomised controlled trials, prophylactic radiotherapy is ineffective in preventing PTMs in unselected patients with mesothelioma. Future studies should explore whether there is any benefit of this treatment in specific patient subgroups—eg, those not receiving chemotherapy and those with epithelioid-only tumour subtypes.

The SMART trial aimed to establish whether immediate radiotherapy given within 42 days of a large-bore pleural intervention reduces the incidence of PTMs developing in mesothelioma, compared with deferred radiotherapy delivered only when PTMs develop. Additionally, we aimed to assess the differences in pain scores, analgesia requirements, quality of life, adverse events, and survival between the treatment groups.

## Methods

### Study design and participants

The SMART trial was a multicentre, open-label, phase 3 randomised controlled trial that recruited consecutive patients from 22 hospitals in the UK (appendix). We recruited patients who had a histocytologically proven diagnosis of malignant pleural mesothelioma and had undergone (in the previous 35 days) an open pleural biopsy, surgical thoracotomy or video-assisted thoracoscopic surgery, local anaesthetic thoracoscopy, large-bore chest tube insertion (≥20 French [Fr] inserted by either a Seldinger technique or blunt dissection), or an indwelling pleural catheter insertion. The 35-day cutoff was extended from 28 days to improve patient recruitement after a protocol amendment accepted by the regional ethics committee on Aug 1, 2012.

Patients were ineligible if they were younger than 18 years, had an expected survival of less than 4 months, were pregnant or lactating, were unable to give informed consent or comply with the protocol, had had previous radiotherapy resulting in an unacceptable overlap with the proposed treatment field, had no access to a telephone, or had a clinically palpable nodule of 1 cm or larger in diameter within 7 cm of the margins of the procedure site at the initial trial visit.

The trial was sponsored by North Bristol National Health Service (NHS) Trust and coordinated by the Respiratory Research Unit at North Bristol NHS Trust. The trial and subsequent protocol amendments were approved by the South Central (Southampton B) Ethics Committee of the UK National Research Ethics Service (REC: 11/SC/0408). There was no data monitoring committee for the trial. The full protocol^18^ was published before the completion of trial follow-up. All patients provided written informed consent before recruitment.

### Randomisation and masking

We randomly assigned patients (1:1) to receive either immediate prophylactic radiotherapy (within 42 days of the pleural intervention) or deferred radiotherapy (given if the patient subsequently developed a PTM). Treatment allocation was done over the telephone by the Oxford Respiratory Trials Unit, operated by staff independent of the study. The randomisation sequence was generated with a validated, online randomisation service (Sealed Envelope, London, UK). Minimisation with a random component was used to reduce the baseline differences between the two groups. The three minimisation factors were histological subtype of mesothelioma (epithelioid only *vs* other), pleural procedure (indwelling pleural catheter *vs* other), and nature of procedure (ie, open pleural biopsy, thoracotomy, or video-assisted thoracoscopic surgery *vs* non-surgical procedure).

Patients and clinicians were not masked to treatment allocation; therefore, outcome assessments were unblinded. Data analysis was blinded from treatment allocation. The trial database was held at The Oxford Respiratory Trials Unit.

### Procedures

Patients allocated to the immediate radiotherapy group received 21 Gy in three fractions over 3 working days, initiated within 42 days of the procedure for which the patient had been randomised. The target volume encompassed the chest drain, surgical sites or scars, and, in the case of indwelling pleural catheters, the whole of the catheter tract and skin exit site, with at least a 3 cm margin. The volume treated needed to have been acceptable to the treating clinical oncologist, and the treatment diameter and width needed to have been at least 7 cm. Further details of the radiotherapy protocol can be found in the published protocol.^18^ The quality assurance programme for the trial is detailed in the appendix.

Patients in the deferred radiotherapy group received radiotherapy only if they were diagnosed with a PTM during trial follow-up. If a PTM was identified, patients received 21 Gy of radiotherapy in three fractions over 3 working days, delivered within 35 days of PTM diagnosis. The target volume encompassed the nodule with at least a 2 cm margin, and the treatment volume needed to have been acceptable to the treating clinical oncologist.

All patients were followed up monthly until death or for 12 months. Patients visited the hospital at randomisation and at 1, 3, 6, 9, and 12 months after randomisation. Each hospital visit included a focused history (detailing radiotherapy toxicity, mesothelioma treatments received, analgesia use, and health-care use) and a chest wall examination by two independent assessors to identify PTMs and radiation toxicity. Patients also completed quality-of-life questionnaires (European Organization for Research and Treatment of Cancer Quality-of-Life Questionnaire—Core 30 [QLQ-C30] and EuroQoL-5D [EQ-5D]), a visual analogue scale score (on a scale of 0–100 mm, with no pain at 0 mm and worst possible pain at 100 mm) for chest pain, and questionnaires about their experience of radiotherapy or the development of a PTM (if applicable). In the months that patients were not seen in person (ie, months 2, 4, 5, 7, 8, 10, and 11), they instead received a telephone call from a research nurse to enquire about symptoms at the intervention site. They also completed a chest pain visual analogue scale score. If problems were identified during a telephone consultation, a clinic appointment was arranged.

Patients recruited at Bristol and Oxford were offered the opportunity to discuss their experience of the trial in a semi-structured, qualitative interview 6 months after trial entry.^18^ Common themes from analysis of these interviews will be identified and reported separately.

Patients were removed from the study only if they withdrew consent for ongoing trial follow-up.

### Outcomes

The primary endpoint was the incidence of PTM within 12 months from randomisation within the intention-to-treat population. A PTM was defined as a clinically palpable nodule of at least 1 cm in diameter that was within 7 cm of the margins of the pleural intervention site, as confirmed by two independent assessors (including at least one doctor who made a clinical diagnosis of tract metastasis). Disagreement was resolved by consensus. For patients who died or were lost to follow-up, their last known outcome was used for the analysis.

The predefined secondary outcomes were a per-protocol analysis of the primary outcome (excluding patients with major protocol violations); summary chest pain visual analogue scale scores from randomisation to 12 months after randomisation; summary quality-of-life score (as measured by QLQ-C30 and EQ-5D) from randomisation to 12 months after randomisation; analgesia requirements; the size, symptom severity, and time to development of PTM; incidence and severity of radiotherapy toxicity (according to the Radiation Therapy Oncology Group [RTOG] grading system^19^); the number of serious adverse events related to radiotherapy or a PTM; overall survival in days from randomisation to death; the health economics of immediate and deferred radiotherapy; and the identification of emergent themes pertaining to patient experience from the semi-structured interviews.

### Statistical analysis

The sample size calculation was based on internal audit data from our regional mesothelioma multidisciplinary team (unpublished data) of PTM rates in those undergoing large-bore procedures and the published literature that was available at the time of the grant application. Assuming a PTM incidence of 2% in the immediate radiotherapy group and 15% in the delayed radiotherapy group, 185 patients were required to show a difference between treatment groups with 90% power (two-sided p=0·05), allowing for a 3% loss to follow-up. Results from various power modelling scenarios showed that the recruitment of 203 patients would allow adequate power with varied control and intervention event rates.^18^ The study was powered to address the primary outcome only, and not the secondary endpoints. The full analysis plan was published before any data assessment or database lock.^18^

We analysed the primary outcome in the intention-to-treat population, using a Fisher's exact test to compare PTM incidence between the treatment groups. We did several predefined subgroup analyses for the primary outcome, including analyses split by type of pleural intervention, histological subtype, survival to 6 months, and receipt of chemotherapy.^18^ In our secondary, predefined, per-protocol analysis, we included only patients with no major protocol violations, defined as failure to give immediate radiotherapy to those randomised to this treatment group, failure to meet the eligibility criteria for the study, or a major violation to the immediate radiotherapy protocol—specifically, giving less than 21 Gy in three fractions, giving radiotherapy more than 42 days after the pleural intervention, or using a radiotherapy field size less than that stipulated in the protocol.

Full details of the analysis of the secondary outcomes can be found in the statistical analysis plan.^18^ Where numbers allowed, the analyses were adjusted for the minimisation factors and the parameter measured at baseline (if the data were continuous). For longitudinal data, an area under the curve was calculated using the trapezium rule, and this was divided by the duration of follow-up to give a summary score, which was used for the analysis. Time-to-event data were analysed using the Kaplan-Meier method. Hazard ratios and 95% CIs were calculated by fitting a Cox model. Safety outcomes were assessed in all patients who received radiotherapy. Safety, toxicity, and patient experience outcomes were reported descriptively. Only serious adverse events related to a PTM or radiotherapy were reported, as per the original trial protocol. The incremental cost-effectiveness ratio was calculated as the difference in mean costs (immediate radiotherapy minus deferred radiotherapy) divided by the difference in mean quality-adjusted life-years (QALYs; immediate radiotherapy minus deferred radiotherapy).

Stata (version 14) was used for statistical analyses. This study was registered with ISRCTN, number ISRCTN72767336.

### Role of the funding source

The funders had no role in the study design, data collection, data analysis, data interpretation, or writing of the report. The corresponding author had full access to all the data in the study and had final responsibility for the decision to submit for publication.

## Results

**Figure 1:**
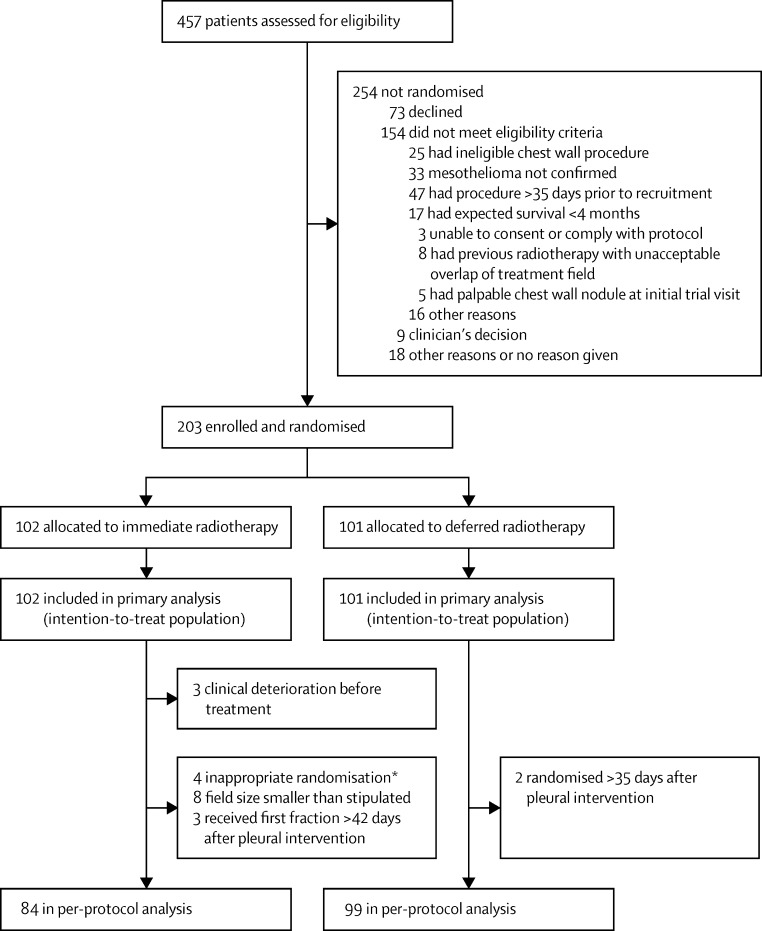
Trial profile *One patient randomised after CT-guided biopsy and more than 35 days after the pleural intervention; three patients randomised more than 35 days after the pleural intervention.

Between Dec 23, 2011, and Aug 4, 2014, we assessed 457 patients for eligibility, and 203 patients were subsequently randomised and included in the primary analysis (figure 1). Baseline characteristics between the two groups were well matched, with no substantial differences identified between the groups (table 1). The number of patients followed up until death or for 12 months was 97 (95%) in the immediate radiotherapy group (three [3%] did not attend and two [2%] withdrew) and 94 (93%) in the delayed radiotherapy group (six [6%] did not attend and one [1%] withdrew). The median duration of follow-up was 343 days (IQR 201–370) in the immediate radiotherapy group and 349 days (212–370) in the deferred radiotherapy group. In the overall study population, 91 (45%) patients were randomised following a video-assisted thoracoscopic surgery, 74 (36%) after a local anaesthetic thoracoscopy, 25 (12%) following an indwelling pleural catheter insertion, nine (4%) after a thoracotomy, and three (1%) following large-bore chest drain insertion (table 1). One patient in the immediate radiotherapy group was randomised in error following a CT-guided pleural biopsy but was included in the primary analysis on an intention-to-treat basis.

99 of 102 patients in the immediate radiotherapy group received the radiotherapy as assigned (three deteriorated clinically between randomisation and treatment precluding irradiation; figure 1). All 99 patients received radiotherapy of 21 Gy in three fractions, delivered using 6–18 meV electrons (85 [87%]), Kv photons (11 [11%]), Mv photons (two [2%]), or unknown (one [1%]; appendix). 97 (98%) patients were treated with a single direct beam, one patient was treated with parallel pair tangents, and one had missing data. 60 (61%) had bolus administered, 38 (38%) had no bolus, and one had missing data. Eight patients had an immediate radiotherapy field size smaller than that stipulated in the protocol and three had the first fraction of immediate radiotherapy given more than 42 days after the randomised procedure (appendix).

Nine of 16 patients in the deferred radiotherapy group who developed a PTM received radiotherapy according to the protocol (appendix). Two patients were too unwell for radiotherapy when the PTM was diagnosed, one patient declined radiotherapy, two patients had a PTM diagnosed at their final trial visit and therefore completed trial follow-up before radiotherapy could be given, and two had missing data.

The other treatments received by patients during their trial follow-up are listed in the appendix. 119 (59%) of 203 patients received first-line pemetrexed–platinum chemotherapy after trial entry, and 11 (5%) of 203 had second-line chemotherapy (appendix).

The primary, intention-to-treat analysis revealed no significant difference between the proportion of patients who developed PTMs in the immediate and deferred radiotherapy groups (nine [8·8%] of 102 patients *vs* 16 [15·8%] of 101 patients respectively; OR 0·51 [95% CI 0·19–1·32]; p=0·14; table 2). The results of the predefined subgroups are shown in table 3.

183 patients were included in the per-protocol analysis (figure 1). Reasons for exclusions were: six patients were inappropriately randomised (one patient in the immediate radiotherapy group had CT-guided biopsy and was randomised more than 35 days after the pleural intervention, and five patients [three in the immediate radiotherapy group and two in the deferred radiotherapy group] were randomised more than 35 days after the pleural intervention); three patients did not have the assigned immediate radiotherapy because of clinical deterioration; eight had an immediate radiotherapy field size smaller than that stipulated in the protocol; and three had the first fraction of immediate radiotherapy given more than 42 days after their pleural intervention. Four patients in the immediate radiotherapy group who developed a PTM were excluded from the per-protocol analysis (in three cases, the field size was too small and one patient was randomised 36 days after the pleural intervention; appendix).

**Figure 2:**
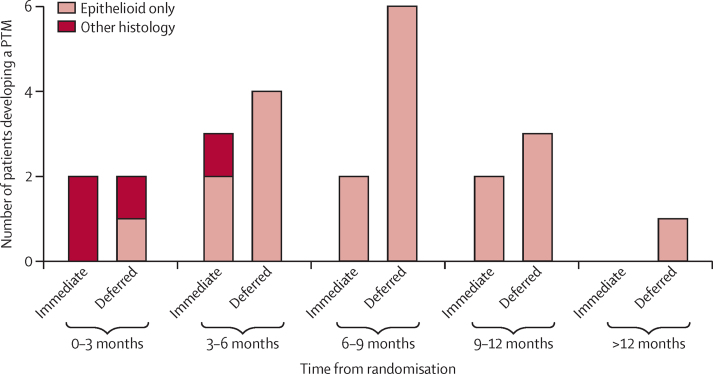
Time to development of PTM, by treatment group and histological subtype PTM=procedure-tract metastasis.

The secondary per-protocol analysis showed that PTM incidence was significantly lower in the immediate radiotherapy group than in the deferred radiotherapy group (table 2). Most PTMs were identified more than 6 months from the pleural intervention procedure after which the patients were randomised (figure 2). No difference was seen in the time to development of PTM from randomisation between the treatment groups (p=0·34; table 2). Nine patients in the immediate radiotherapy group had PTMs (mean size of PTMs at diagnosis 3·1 cm [SD 2·9]), and 16 patients in the deferred radiotherapy group had PTMs (4·6 cm [2·6]; p=0·083). There were insufficient data for a formal analysis of how the size of the nodules changed over time. The minimum distance between the procedure site and the edge of the PTM was longer in the immediate radiotherapy group (mean 4·6 cm [SD 2·6]) than in the deferred radiotherapy group (1·3 cm [1·8]; p=0·0049). Four of the nine patients who developed a PTM in the immediate radiotherapy group did so more than 2 cm from the immediate radiotherapy field. In addition to the 25 patients who developed a PTM within 7 cm of the site of pleural intervention procedure (meeting the primary endpoint criteria), a further four patients in the immediate radiotherapy group developed an ipsilateral chest wall nodule more than 7 cm from the procedure site. No significant difference was identified in the total number of patients developing a chest wall nodule anywhere on the ipsilateral hemithorax (table 2).

No difference was reported in chest pain, as assessed by visual analogue scale scores, in the two treatment groups at baseline and during trial follow-up (Table 1, Table 4). Additionally, no difference was seen between the groups in terms of analgesia use, as measured by oral morphine-equivalent doses at baseline, or summary scores during trial follow-up (Table 1, Table 4).

Of the 25 patients who developed PTM, no differences were identified in terms of the summary visual analogue scale scores or morphine-equivalent doses between the treatment groups (table 2). The numbers were too small for a formal survival analysis from the time of PTM diagnosis to death. Results of the chest wall lump questionnaire, completed by patients who developed PTMs, also revealed no differences in terms of patient experience between the treatment groups (appendix). Only eight patients (4% of 203 patients in the study; 32% of 25 patients who developed a PTM) had PTMs that were tender at the time of identification (table 2). The number needed to treat to prevent one painful PTM was 25·1. The proportion of PTMs that were painful was similar in the immediate radiotherapy group (two [22%] of nine) and in the deferred radiotherapy group (six [38%] of 16; odds ratio 0·48 [95% CI 0·04–3·94]; p=0·66; table 2).

Quality of life of the two groups—as measured by the global health status, fatigue, pain, and appetite loss subscales of the QLQ-C30—was similar at baseline and during trial follow-up, both in terms of interval change from baseline to 1 month and in terms of the overall summary score during trial follow-up (Table 1, Table 4). No difference in quality of life was identified at baseline or during trial follow-up by the summary scores derived from the EQ-5D utility scores (table 4).

**Figure 3:**
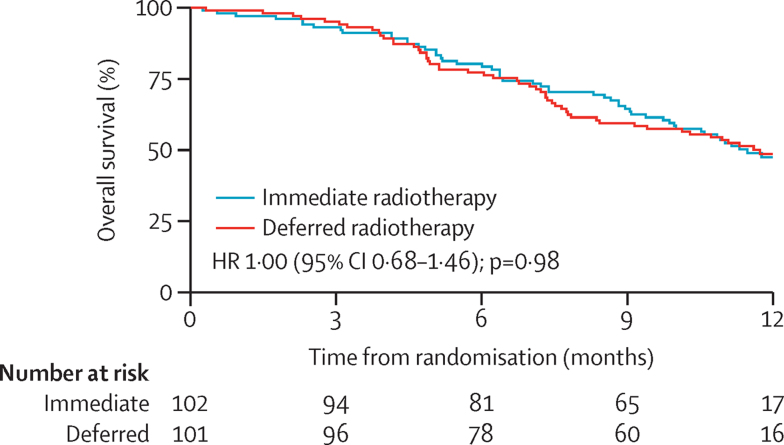
Overall survival from randomisation HR=hazard ratio.

Median overall survival from randomisation was 357 days (95% CI not estimable) in the immediate radiotherapy group and 365 days (not estimable) in the deferred radiotherapy group (figure 3).

Immediate radiotherapy was generally well tolerated both in terms of side-effects and in terms of patient experience (table 5; appendix). There were no treatment-related deaths or withdrawals. Only one serious adverse event related to a PTM or radiotherapy was reported: one patient in the immediate radiotherapy group developed a painful PTM within the radiotherapy field that required hospital admission for symptom control 8 months after randomisation (table 5). Among patients who received immediate radiotherapy, common adverse events were grade 1 skin toxicity and tiredness or lethargy within 3 months of receiving radiotherapy (table 5). The relative incidence of indwelling pleural catheter complications was similar in the two groups (appendix). Of 93 patients in the immediate radiotherapy group who completed the patient experience questionnaire, 26 (28%) patients said that they found attending radiotherapy at least a little inconvenient, 27 (29%) reported that radiotherapy interfered with their usual activities, and 27 (29%) said that radiotherapy affected their quality of life (appendix). However, 84 (90%) patients said that they found attending radiotherapy at least a little reassuring (appendix). Five patients in the deferred radiotherapy group completed the post-radiotherapy questionnaire, and their experiences were comparable to those of the immediate radiotherapy group (appendix).

No significant or meaningful differences were seen in mean total cost (£5475 [SD 703] in the immediate radiotherapy group *vs* £5473 [773] in the deferred radiotherapy group) or in mean QALYs (0·504 [SD 0·03] *vs* 0·516 [0·03]). The point estimate of the incremental cost-effectiveness ratio for immediate radiotherapy compared with deferred radiotherapy was –£85·19 per QALY.

## Discussion

Results from the SMART trial showed that, for patients with malignant pleural mesothelioma, treatment with prophylactic radiotherapy after large-bore pleural interventions does not reduce the incidence of PTM and confers no benefits in terms of symptom control, analgesia use, survival, or quality of life. To our knowledge, the SMART trial is the largest trial investigating this research question and the first to fully assess the important patient-centred outcomes of pain and quality of life.

The overall incidence of PTM in this study was low, with 93 (91%) patients in the immediate radiotherapy group and 85 (84%) in the deferred radiotherapy group not developing a PTM during trial follow-up. No significant difference in the incidence of PTM was seen between the treatment groups, despite our study being the largest so far and having recruited more patients than the three previous randomised controlled trials^5^, ^6^, ^7^ combined. This finding, along with the absence of any patient-centred, symptomatic benefit conferred by immediate radiotherapy, suggests that even if prophylactic radiotherapy was mildly efficacious if given to a large enough number of patients (number needed to treat to prevent a painful PTM 25·1), the patient-derived benefit would be no better than careful clinical follow-up and treatment should a nodule develop.

Some potentially interesting findings from the secondary subgroup analyses might warrant further investigation. The secondary per-protocol analysis, which excluded patients with serious protocol deviations, showed a difference in PTM incidence between the treatment groups. This finding highlights the possibility that if adequate radiotherapy is given according to our protocol, the rate of PTM might be reduced, but the clinical relevance of this and whether reliable delivery of such a protocol in real-world settings is feasible (in view of the number of protocol deviations in this trial) are unclear. Importantly, however, the lack of difference between the treatment groups in terms of any of the patient-centred outcomes (including pain and quality of life) and the low rate of occurrence of symptomatic nodules suggest that patients were not disadvantaged by an active surveillance strategy and prompt administration of radiotherapy in the event of a nodule being detected.

Two potential signals were identified in the predefined subgroup analyses. The first was a suggestion that immediate radiotherapy was effective in reducing PTMs in the subgroup of patients with epithelioid-only histological tumour subtypes, which is likely to reflect the improved survival of this subgroup and hence an increased duration of potential benefit from the treatment. Second, in the subgroup of patients who did not receive chemotherapy after trial enrolment, those who received immediate radiotherapy had a lower PTM rate than had those in the deferred radiotherapy group. This finding suggests potential benefits in terms of prevention of locoregional recurrence from chemotherapy in addition to the well established systemic benefits. It might also explain the results of a large retrospective case series^20^ supporting a role for prophylactic irradiation of tracts, in which the proportion of patients receiving chemotherapy was significantly lower in patients who did not receive immediate radiotherapy than in those who did. The results of the PIT study,^21^ a randomised controlled trial assessing prophylactic irradiation of tracts in mesothelioma, which is in its follow-up period at present, are awaited with interest. These results will allow combination of the available data from published studies to gain further insights into the role of prophylactic radiotherapy in mesothelioma, particularly in these potentially relevant subgroups identified in our study.

Overall, the rate of symptomatic PTM in this study was low. Eight (4%) of 203 patients in the study had a tender nodule at presentation, and eight (32%) of the 25 patients who developed a PTM had a tender nodule, which is similar to the proportions in a previous study.^6^ No difference was identified between the treatment groups, again suggesting that prophylactic radiotherapy does not affect the development of symptoms from PTMs in this population. Only one patient, who was in the immediate radiotherapy group, required hospital admission for symptom control relating to a PTM.

Complication rates of indwelling pleural catheters did not seem to be affected by radiotherapy, suggesting that these catheters are a safe treatment in this context, although again we did not identify any benefits of delivering prophylactic radiotherapy in this subgroup of patients. However, it is reassuring that the catheters themselves were not adversely affected by radiotherapy treatment, suggesting that palliative radiotherapy could be safely administered to the region of an indwelling pleural catheter if necessary.

Immediate prophylactic radiotherapy in this population was not cost-effective, compared with deferred radiotherapy for symptomatic treatment. If we assume that publicly funded health-care systems (eg, NHS in the UK) are willing to pay £30 000 for incremental health gains (ie, an additional QALY), then immediate radiotherapy compared with deferred radiotherapy in patients with malignant mesothelioma following large-bore chest procedure is estimated to be cost-effective only 40% of the time (data to be published in full in a separate paper). However, radiotherapy was generally well tolerated and the frequency of skin toxicity was low, despite the larger field size used in this trial than in previous studies. Most patients who received immediate radiotherapy found it reassuring, although they did report some inconvenience associated with attendance for the radiotherapy sessions.

This study has several strengths. To our knowledge, it is the largest study so far and was suitably powered to investigate this important research question in mesothelioma. It was designed to be a pragmatic trial to assess prophylactic radiotherapy in mesothelioma in a real-world setting. We assessed several important patient-centred secondary endpoints that have not been previously studied. We followed up patients until death or for 12 months, ensuring that all PTMs were carefully identified by independent examinations by two clinicians at each clinic visit, which is essential in view of the fact that many PTMs were identified in the later months of trial follow-up. The SMART trial is also the first to investigate the role of prophylactic radiotherapy since effective systemic chemotherapy has become available for mesothelioma and to assess its role in the context of indwelling pleural catheter use.

Potential limitations include the absence of blinding of participants or assessors, which would not have been possible within the trial budget. The failure rate of screening was high, predominantly because patients did not meet the trial's eligibility criteria, which were all deemed important factors to ensure that the results were valid and robust. The number of protocol deviations, specifically those related to the delivery of immediate radiotherapy, could also be viewed as a limitation of this study. However, these deviations in compliance with the protocol are likely to reflect real-world variation in practice and might be even more substantial outside the trial setting. The field size of radiotherapy was selected on the basis of results from previous studies and what was deemed reasonable to minimise radiotherapy toxicity; however, it could be argued that an even larger, more superiorly configured field might have been more effective.^22^ However, whether these more distant nodules represent true PTMs or local tumour invasion unrelated to the previous pleural interventions is impossible to distinguish, and selection of a practical and realistic radiotherapy protocol was deemed vital for the purposes of this study to make the results universally applicable. Few patients in the SMART trial had undergone large surgical procedures (eg, thoracotomies), and hence it is not possible to give a specific recommendation regarding prophylactic radiotherapy in this group of patients.

Overall, our data do not support the use of routine prophylactic radiotherapy after large-bore pleural interventions for all patients with mesothelioma. Although some suggestion of benefit was identified in specific patient subgroups, the applicability of these findings is limited by the small numbers. Therefore, more data from randomised controlled trials are needed before the use of prophylactic radiotherapy in these contexts can be fully established. No significant difference was identified in terms of PTM rate, chest pain, quality of life, analgesia requirements, or survival, and immediate radiotherapy was not cost-effective. Patients were not symptomatically disadvantaged by careful clinical follow-up and later radiotherapy should a PTM develop. Our findings do not support routine use of prophylactic irradiation of tracts in mesothelioma, provided that the patient receives regular clinical follow-up to ensure symptoms are identified and treated early.

## Figures and Tables

**Table 1:** Baseline characteristics

		**Immediate radiotherapy (n=102)**	**Deferred radiotherapy (n=101)**
Sex			
	Male	91 (89%)	90 (89%)
	Female	11 (11%)	11 (11%)
Age (years)		70 (66–76)	70 (66–77)
Time from diagnosis to randomisation (days)		21 (14–25)	20 (13–27)
Mean time from pleural intervention to randomisation (days)		22·8 (7·7)	22·2 (8·1)
WHO performance score			
	0	29 (28%)	34 (34%)
	1	61 (60%)	53 (52%)
	2	9 (9%)	11 (11%)
	3	3 (3%)	3 (3%)
Karnofsky performance score		90 (80–90)*	80 (80–90)
Body-mass index		26·2 (4·3)†	26·5 (3·3)†
Extrathoracic spread on imaging			
	Yes	4 (4%)	2 (2%)
	No	81 (79%)	80 (79%)
	Unknown	17 (17%)	19 (19%)
Histological subtype			
	Epithelioid only	71 (70%)	71 (70%)
	Sarcomatoid	8 (8%)	8 (8%)
	Biphasic (mixed)	19 (19%)	18 (18%)
	Desmoplastic	4 (4%)	0
	Other	0	4 (4%)
Basis for diagnosis			
	Pleural fluid cytology	0	3 (3%)
	Pleural biopsy	102 (100%)	98 (97%)
Side of disease			
	Left	32 (31%)	40 (40%)
	Right	69 (68%)	61 (60%)
	Bilateral	1 (1%)	0
Smoking status			
	Current	6 (6%)	7 (7%)
	Former	59 (58%)	54 (53%)
	Never	37 (36%)	40 (40%)
Comorbidities			
	Respiratory disease	5 (5%)	11 (11%)
	Cardiac disease	9 (9%)	12 (12%)
	Chronic renal failure	3 (3%)	3 (3%)
	Diabetes	15 (15%)	14 (14%)
	Steroid use	6 (6%)	5 (5%)
Symptomatic pleural effusion at presentation		91 (89%)	90 (89%)
Chest pain at presentation		36 (35%)	37 (37%)
Previous pleurodesis		54 (53%)	68 (67%)
Type of pleural intervention			
	Large-bore chest drain insertion	1 (1%)	2 (2%)
	Local anaesthetic thoracoscopy	38 (37%)	36 (36%)
	Thoracotomy	3 (3%)	6 (6%)
	Video-assisted thoracoscopic surgery	45 (44%)	46 (46%)
	Indwelling pleural catheter insertion	14 (14%)	11 (11%)
	Other	1 (1%)‡	0
Number of pleural puncture sites			
	1	69 (68%)	68 (67%)
	2	27 (26%)	26 (26%)
	≥3	6 (6%)	6 (6%)
	Unknown	0	1 (1%)
Previous chemotherapy received for mesothelioma		3 (3%)	6 (6%)
Previous radiotherapy received for mesothelioma		1 (1%)	2 (2%)
Oral morphine-equivalent dose (mg)		0 (0–0)	0 (0–0)
Quality of life§			
	QLQ-C30 global health status subscale	66·7 (50·0–83·3), n=98	66·7 (50·0–83·3), n=96
	QLQ-C30 physical functioning subscale	80·0 (53·3–93·3), n=98	76·7 (60·0–86·7), n=96
	QLQ-C30 pain subscale	16·7 (0·0–33·3), n=98	16·7 (0·0–33·3), n=97
	EQ-5D utility score	0·79 (0·69–0·85), n=100	0·78 (0·62–0·85), n=98
Chest pain (visual analogue scale scores)¶			
	On average how much chest pain have you felt today?	5·0 (0·0–17·0)	4·0 (0·5–18·5)
	How much has chest pain bothered you today?	3·3 (0·0–13·7)	3·0 (0·0–14·8)
	On average how much pain have you felt today from the site of your previous chest wall procedure?	3·0 (0·0–10·0)	2·8 (0·5–15·8)
	How much has pain from the site of your previous chest wall procedure bothered you today?	1·5 (0·0–8·0)	3·0 (0·5–15·0)

Data are n (%), median (IQR), or mean (SD). QLQ-C30=European Organization for Research and Treatment of Cancer Quality-of-Life Questionnaire—Core 30. EQ-5D=EuroQoL-5D.

* n=101; one patient had missing data for Karnofsky performance score at baseline.

† n=98 in the immediate radiotherapy group and n=97 in the delayed radiotherapy group because the data were not recorded at baseline for the remaining patients.

‡ CT-guided biopsy; this patient was randomised in error.

§ Quality-of-life scores were derived from two separate questionnaries (QLQ-C30 and EQ-5D), and not all patients completed both at baseline; not all patients completed individual questions necessary to calculate a certain QLQ-C30 subscale.

¶ n=97 in the delayed radiotherapy group because the remaining patients did not complete baseline visual analogue scale scores.

**Table 2:** PTM development

		**Immediate radiotherapy**	**Deferred radiotherapy**	**Odds ratio (95% CI)**	**p value**
Number of patients developing a PTM					
	Intention-to-treat analysis	9/102 (9%)	16/101 (16%)	0·51 (0·19–1·32)	0·14
	Per-protocol analysis	5/84 (6%)	16/99 (16%)	0·33 (0·09–1·00)	0·037
Number of patients developing a painful PTM		2/102 (2%)	6/101 (6%)	0·32 (0·03–1·84)	0·17
Time to development of PTM from randomisation (days)		179 (126–221)	224 (136–285)	NA*	0·34
Summary chest pain visual analogue scale score from diagnosis of PTM to 12 months after randomisation†					
	On average how much chest pain have you felt today?	26·2 (10·3–53·7)	18·8 (5·5–32·2)	NA*	0·74
	On average how much has chest pain bothered you today?	24·5 (9·7–51·9)	16·4 (4·4–49·7)	NA*	0·92
	On average how much pain have you felt today from the site of your previous chest wall procedure?	9·9 (7·1–28·3)	15·4 (8·2–42·4)	NA*	0·63
	On average how much has pain from the site of your previous chest wall procedure bothered you today?	12·3 (7·0–28·5)	13·0 (6·4–42·9)	NA*	0·77
Summary morphine-equivalent dose from diagnosis of PTM to 12 months after randomisation‡		12·6 (1·4–33·5)	16·0 (5·0–45·0)	NA*	0·53
Number of patients developing a chest wall nodule anywhere on the ipsilateral hemithorax		13/102 (13%)	16/101 (16%)	0·78 (0·32–1·84)	0·55

Data are n/N (%) or median (IQR), unless otherwise specified. PTM=procedure-tract metastasis. NA=not applicable.

* Because of the small number of patients, the groups were compared by Mann Whitney U tests; therefore, no treatment effect was obtained.

† n=8 in the immediate radiotherapy group and n=9 in the deferred radiotherapy group, because there were insufficient data after PTM development to calculate an area under the curve for the remaining patients.

‡ n=7 in the immediate radiotherapy group; n=10 in the deferred radiotherapy group.

**Table 3:** Subgroup analyses for the primary outcome

		**Median time to development of PTM from randomisation (days)**	**Number of patients developing a PTM**			
			Immediate radiotherapy	Deferred radiotherapy	Odds ratio (95% CI)	p value
Pleural intervention procedure type						
	Large-bore chest drain insertion (≥20 Fr)	NA	0/1 (0%)	0/2 (0%)	NA	NA
	Local anaesthetic thoracoscopy	187·5 (140–265)	4/38 (11%)	4/36 (11%)	0·94 (0·16–5·51)	1·00
	Thoracic surgery (thoracotomy or video-assisted thoracic surgery)	221 (146–290)	4/48 (8%)	9/52 (17%)	0·43 (0·09–1·71)	0·24
	Indwelling pleural catheter insertion	136·5 (80·5–206·5)	1/14 (7%)	3/11 (27%)	0·21 (0·00–3·27)	0·29
Tumour subtype						
	Epithelioid only	221 (158–285)	6/71 (8%)	15/72 (21%)	0·35 (0·11–1·04)	0·057
	Other	32·5 (23·0–94·5)	3/31 (10%)	1/29 (3%)	3 (0·22–163)	0·61
Patients followed-up for ≥6 months		221 (158–285)	8/80 (10%)	13/76 (17%)	0·54 (0·18–1·51)	0·24
Chemotherapy after trial entry						
	Yes	221 (126–266)	7/56 (13%)	8/64 (13%)	1·00 (0·29–3·41)	1·00
	No	163 (101–304)	2/46 (4%)	8/37 (22%)	0·16 (0·02–0·93)	0·021

Data are median (IQR), or n/N (%), unless otherwise specified. PTM=procedure-tract metastasis. NA=not available.

**Table 4:** Visual analogue scale scores, quality-of-life variables, and morphine-equivalent doses

		**Number included in analysis**		**Median number of timepoints used to generate summary score**		**Summary score**		Difference in means*	**p value**
		Immediate radiotherapy	Deferred radiotherapy	Immediate radiotherapy	Deferred radiotherapy	Immediate radiotherapy	Deferred radiotherapy		
Summary visual analogue scale score									
	On average how much chest pain have you felt today?	93	96	10 (6 to 12)	10 (5 to 12)	12·0 (3·3 to 23·3)	9·0 (2·9 to 27·0)	0·6 (−3·3 to 4·6)	0·75
	On average how much has chest pain bothered you today?	93	96	10 (6 to 12)	10 (5 to 12)	9·1 (2·7 to 20·6)	7·8 (2·6 to 24·1)	0·8 (−3·1 to 4·6)	0·70
	On average how much pain have you felt today from the site of your previous chest wall procedure?	93	95	10 (6 to 12)	10 (5 to 12)	6·9 (2·4 to 16·4)	5·3 (2·2 to 16·8)	1·0 (−2·3 to 4·2)	0·56
	On average how much has pain from the site of your previous chest wall procedure bothered you today?	93	95	10 (6 to 12)	10 (5 to 12)	5·5 (1·2 to 15·1)	5·5 (2·1 to 17·7)	0·5 (−2·7 to 3·6)	0·76
QLQ-C30 summary score									
	Global health status	91	92	5 (3 to 6)	5 (3 to 6)	59·6 (45·8 to 70·8)	61·6 (43·1 to 74·9)	−1·2 (−5·2 to 2·8)	0·55
	Physical functioning	91	92	5 (3 to 6)	5 (3 to 6)	71·0 (54·8 to 83·7)	70·3 (49·5 to 84·4)	−0·1 (−3·1 to 2·9)	0·94
	Pain	91	93	5 (3 to 6)	5 (3 to 6)	22·8 (8·7 to 35·1)	16·7 (6·7 to 35·9)	1·3 (−3·1 to 5·6)	0·57
EQ-5D summary score (time trade-off scores)		94	95	5 (3 to 6)	5 (3 to 6)	0·72 (0·56 to 0·83)	0·72 (0·58 to 0·82)	−0·02 (−0·06 to 0·02)	0·38
Mean change in QLQ-C30 scores from randomisation to 1 month									
	Global health status	83	87	NA	NA	6·2 (21·0)	5·3 (24·3)	0·8 (−5·5 to 7·0)	0·81
	Physical functioning	83	88	NA	NA	5·7 (12·6)	4·5 (12·7)	0·9 (2·8 to −4·7)	0·62
	Fatigue	83	88	NA	NA	−3·2 (23·1)	−2·6 (20·8)	0·0 (−5·5 to 5·5)	1·00
	Pain	84	88	NA	NA	−0·6 (22·2)	−3·2 (23·0)	2·8 (−3·3 to 9·0)	0·36
	Appetite loss	83	88	NA	NA	2·0 (30·1)	−2·7 (32·1)	1·9 (−5·5 to 9·2)	0·62
Morphine-equivalent dose summary score		102	101	5 (4 to 6)	5 (4 to 6)	2·7 (0 to 17·3)	1·9 (0 to 10·5)	NA†	0·50

Data are n, median (IQR), or mean (SD). QLQ-C30=European Organization for Research and Treatment of Cancer Quality-of-Life Questionnaire—Core 30. EQ-5D=EuroQoL-5D. NA=not applicable.

* Adjusted difference in mean summary scores (immediate radiotherapy group – deferred radiotherapy group).

† Non-parametric comparison was used because of skewed data, so no difference in means was generated.

**Table 5:** Adverse events related to radiotherapy, according to the trial protocol

		**Within 3 months of receiving radiotherapy**		**>3 months after receiving radiotherapy**	
		Immediate radiotherapy (n=92)*	Deferred radiotherapy (n=5)†	Immediate radiotherapy (n=78)‡	Deferred radiotherapy (n=1)§
Skin toxicity¶					
	Grade 0	38 (41%)	3 (60%)	37 (47%)	1 (100%)
	Grade 1	50 (54%)	2 (40%)	40 (52%)	0
	Grade 2	4 (4%)	0	1 (1%)	0
	Grade 3	0	0	0	0
	Grade 4	0	0	0	0
Subcutaneous tissue toxicity¶‖					
	Grade 0	..	..	76 (97%)	1 (100%)
	Grade 1	..	..	2 (3%)	0
	Grade 2	..	..	0	0
	Grade 3	..	..	0	0
	Grade 4	..	..	0	0
	Grade 5	..	..	0	0
Nausea		10 (11%)	0	3 (4%)	0
Tiredness or lethargy		36 (39%)	2 (40%)	18 (23%)	0
Loss of appetite		1 (1%)	0	0	0
Pain		2 (2%)	0	0	0

Data are n (%). Adverse events in this table relate to either immediate radiotherapy or deferred radiotherapy (given to treat a PTM). PTM=procedure-tract metastasis.

* Ten of 102 patients were excluded (five were too unwell or died before follow-up, one declined follow up, and four had missing data).

† Four of nine patients were excluded (two died before follow-up and two had missing data).

‡ 24 patients were excluded (21 were too unwell or died before follow-up, one declined follow-up, and two had missing data).

§ Eight patients were excluded (four died before follow-up, three had missing data, and one developed PTM too late in the follow-up period to identify late toxicity.

¶ Radiation Therapy Oncology Group grading system.

‖ Subcutaneous tissue toxicity is not in early assessments of the grading system.

## References

[1] Health and Safety Executive (2014). Mesothelioma in Great Britain 2014.

[2] Tan E, Warren N (2009). Projections of mesothelioma mortality in Great Britain.

[3] Agarwal PP, Seely JM, Matzinger FR (2006). Pleural mesothelioma: sensitivity and incidence of needle track seeding after image-guided biopsy versus surgical biopsy. Radiology.

[4] Carmichael J, Degraff WG, Gamson J (1989). Radiation sensitivity of human lung cancer cell lines. Eur J Cancer Clin Oncol.

[5] Boutin C, Rey F, Viallat JR (1995). Prevention of malignant seeding after invasive diagnostic procedures in patients with pleural mesothelioma. A randomized trial of local radiotherapy. Chest.

[6] O'Rourke N, Garcia JC, Paul J, Lawless C, McMenemin R, Hill J (2007). A randomised controlled trial of intervention site radiotherapy in malignant pleural mesothelioma. Radiother Oncol.

[7] Bydder S, Phillips M, Joseph DJ (2004). A randomised trial of single-dose radiotherapy to prevent procedure tract metastasis by malignant mesothelioma. Br J Cancer.

[8] Lee C, Bayman N, Swindell R, Faivre-Finn C (2009). Prophylactic radiotherapy to intervention sites in mesothelioma: a systematic review and survey of UK practice. Lung Cancer.

[9] Nagendran M, Pallis A, Patel K, Scarci M (2011). Should all patients who have mesothelioma diagnosed by video-assisted thoracoscopic surgery have their intervention sites irradiated?. Interact Cardiovasc Thorac Surg.

[10] Ung YC, Yu E, Falkson C (2006). The role of radiation therapy in malignant pleural mesothelioma: a systematic review. Radiother Oncol.

[11] De Ruysscher D, Slotman B (2003). Treatment of intervention sites of malignant pleural mesothelioma with radiotherapy: a Dutch-Belgian survey. Radiother Oncol.

[12] British Thoracic Society Standards of Care Committee (2007). BTS statement on malignant mesothelioma in the UK, 2007. Thorax.

[13] Scherpereel A, Astoul P, Baas P (2010). Guidelines of the European Respiratory Society and the European Society of Thoracic Surgeons for the management of malignant pleural mesothelioma. Eur Respir J.

[14] van Zandwijk N, Clarke C, Henderson D (2013). Guidelines for the diagnosis and treatment of malignant pleural mesothelioma. J Thorac Dis.

[15] Stahel RA, Weder W, Lievens Y, Felip E, Group EGW (2010). Malignant pleural mesothelioma: ESMO Clinical Practice Guidelines for diagnosis, treatment and follow-up. Ann Oncol.

[16] Ettinger DS, Akerley W, Borghaei H (2012). Malignant pleural mesothelioma. J Natl Compr Canc Netw.

[17] Lianes P, Remon J, Bover I, Isla D (2011). SEOM guidelines for the treatment of malignant pleural mesothelioma. Clin Transl Oncol.

[18] Clive AO, Wilson P, Taylor H (2015). Protocol for the surgical and large bore procedures in malignant pleural mesothelioma and radiotherapy trial (SMART Trial): an RCT evaluating whether prophylactic radiotherapy reduces the incidence of procedure tract metastases. BMJ Open.

[19] Cox JD, Stetz J, Pajak TF (1995). Toxicity criteria of the Radiation Therapy Oncology Group (RTOG) and the European Organization for Research and Treatment of Cancer (EORTC). Int J Radiat Oncol Biol Phys.

[20] Froment MA, Frechette E, Dagnault A (2011). Prophylactic irradiation of intervention sites in malignant pleural mesothelioma. Radiother Oncol.

[21] Bayman N, Ardron D, Ashcroft L (2016). Protocol for PIT: a phase III trial of prophylactic irradiation of tracts in patients with malignant pleural mesothelioma following invasive chest wall intervention. BMJ Open.

[22] Hambleton K, Faivre-Finn C, Baldwin D (2009). Prevention of skin metastasis in malignant mesothelioma with prophylactic irradiation of tracts (PIT): is the difference in research evidence due to the discrepancy between the distance from pleural entry point and the skin scar?. Thorax.

