# Supplementary material for: Prophylactic radiotherapy for the prevention of procedure-tract metastases after surgical and large-bore pleural procedures in malignant pleural mesothelioma (SMART): a multicentre, open-label, phase 3, randomised controlled trial
Source: Lancet Oncol. 2016 Aug;17(8):1094–104. doi: 10.1016/S1470-2045(16)30095-X (PMC4961873; doi:10.1016/S1470-2045(16)30095-X)
Supplement: Supplementary appendix [file mmc1.pdf]

THE LANCET Oncology

Supplementary appendix

This appendix formed part of the original submission and has been peer reviewed. We post it as supplied by the authors.

Supplement to: Clive AO, Taylor H, Dobson L, et al. Prophylactic radiotherapy for the prevention of procedure-tract metastases after surgical and large-bore pleural procedures in malignant pleural mesothelioma (SMART): a multicentre, open-label, phase 3, randomised controlled trial. *Lancet Oncol* 2016; published online June 23. http://dx.doi.org/10.1016/S1470-2045(16)30095-X.

Appendix for SMART Trial Manuscript

Recruiting Centres

Principal Investigator	Recruiting Centre	Number of patients recruited
Prof N Maskell	North Bristol NHS Trust	43
Mr A Marchbank	Plymouth Hospitals NHS Trust	18
Dr L Bishop	Portsmouth Hospitals NHS Trust	15
Dr J Pepperell	Taunton and Somerset NHS Foundation Trust	14
Dr N Rahman	Oxford University Hospitals NHS Foundation Trust	13
Dr A Ionescu	Royal Gwent Hospital, Newport	12
Dr E De Winton	Royal United Hospital Bath NHS Trust	12
Dr P Wilson & Dr C Comins	United Hospitals Bristol NHS Foundation Trust	11
Dr A Kerry	Great Western Hospitals NHS Trust	7
Dr S Cooper & Dr Muthukumar	Colchester Hospital University NHS Foundation Trust	7
Dr M Bayne	Poole Hospital NHS Foundation Trust	7
Dr E Toy	Royal Devon and Exeter NHS Foundation Trust	6
Dr P Jenkins	Gloucestershire Hospitals NHS Trust	6
Dr V Vigneswaran	Singleton Hospital, Swansea	5
Dr M Tomlinson	Weston Area NHS Trust	5
Dr J Gildersleve	Royal Berkshire NHS Foundation Trust	5
Dr M Bayne	Dorset County Hospital NHS Foundation Trust	4
Dr N Panakis	Buckinghamshire Healthcare NHS Trust	4
Dr M Button	Nevill Hall Hospital, Abergavenny	3
Dr M Ahmed	The Royal Marsden NHS Foundation Trust	3
Dr C Lewanski	Imperial College Healthcare NHS Trust	2
Dr V Vigneswaran	Withybush Hospital, Haverfordwest	1

Other treatments received by patients during trial follow up

Treatment		Immediate RT n (%). n=102	Deferred RT, n (%). n=101
Chemotherapy	Any chemotherapy	56 (54·9%)	64 (63·4%)
	1st line pemetrexed/platinum chemotherapy	56 (55)	63 (62)
	2nd line chemotherapy	6 (6)	5 (5)
Radiotherapy	For chest wall nodule	1 (1)	10 (10)
	Other palliative reason	1 (1)	4 (4)
	Other	0	1 (1)
Thoracic surgery		0 (0·0%)	4 (4·0%)
Palliative Care involvement		53 (52·0%)	50 (49·5%)
Cordotomy		2 (2·0%)	2 (2·0%)
Pleural interventions		10 (9·8%)	21 (20·8%)
Other clinical trial involvement		4 (4%)	7 (7%)

IPC complications

IPC complications	Immediate radiotherapy arm n (%)	Deferred radiotherapy arm n (%)	*p*-value
Pleural infection	0/29 (0)	2/32 (6)	0·49
IPC Blockage	4/29 (14)	3/32 (9)	0·70
IPC fracture	0/29	0/32	1·00
Local skin cellulitis	2/28 (7)	3/32 (9)	1·00
Drain dislodgment	1/29 (3)	0/32 (0)	0·48
Damage to the IPC plastic	0/28 (0)	0/32	1·00
IPC removal attempted	11/29 (38)	11/32 (34)	0·80
Difficulties removing the IPC	0/10	0/11	1·00

Median overall survival from diagnosis of mesothelioma to death

Median overall survival from diagnosis of mesothelioma was 388 days (95% CI not estimable) in the immediate radiotherapy group and 400 days (not estimable) in the deferred radiotherapy group (from diagnosis hazard ratio 1·02, 95% CI 0·70–1·50; p=0·900).

Radiotherapy received

		Immediate radiotherapy group (n=102)		Deferred radiotherapy group (n=101)
**Total number of patients receiving radiotherapy during trial, n (%)**	Prophylactic radiotherapy	99 (97%)		0
	For nodule within 7cm of randomised procedure site	1		9
	For nodule >7cm from randomised procedure site	0		1*
	Other palliative radiotherapy	1		4
	Other indication	0		1 (post pleurectomy)
**RT modality used, n (%)**		**Prophylactic radiotherapy**	**Other radiotherapy**	
	6-18 MeV Electrons	85	1 (for other palliative reason)	6 (for nodule <7cm from procedure site)
	Kv photons	11	0	2 (for nodule <7cm from procedure site)
	Mv photons	2	1 (nodule <7cm from site)	1 (for nodule <7cm from procedure site)<br>1 (for nodule >7cm from procedure site)*<br>3 (for other palliative reason)
	Not known	1	0	1 (post pleurectomy)<br>1 (for other palliative reason)
**RT protocol deviations, n (%)**	Failure to give radiotherapy when indicated according to the protocol	3 (clinical decline)		7 (2 patients had PTM diagnosed at 12 month follow up, therefore completed trial before radiotherapy given; 2 patients were too unwell to receive radiotherapy at time of PTM diagnosis; 1 patient declined radiotherapy; 2 missing data)
	Radiotherapy given out of the stipulated time-frame	4 (given >42 days after procedure)		1 (given >42 days after PTM diagnosis)
	Radiotherapy field margin smaller than that stipulated in the protocol (ie <7x7cm)	8 (field <7cm in either width or length)		0
	Other major radiotherapy protocol deviation	0		2 (2 patients given 10Gy in 1# to treat PTM)

RT= Radiotherapy; kv= kilovolts; MV= megavolts. * This patient received radiotherapy for a chest nodule >7cm from the pleural intervention site, however, this was not formally identified at clinical examination during a SMART trial visit and hence was not included for the purposes of the trial analysis.

Radiotherapy Quality Assurance (QA) for the trial

Advice regarding the radiotherapy QA for the trial was obtained from the NCRI Radiotherapy Trials QA Group at the start of the trial in January 2012, who reviewed the protocol and case report forms. As the standard radiotherapy technique in the trial was a single field with no CT planning, they advised that the QA requirement was minimal. Based on their recommendations, we undertook the following QA:

1. Obtained evidence of an independent audit measurement from all the centres delivering radiotherapy within the last 5 years for the selected treatment modality for the trial.

2. A data query was raised for any field size smaller than 7x7cm.

3. Audit of compliance with the radiotherapy protocol during recruitment period

Overall the NCRI Radiotherapy Trials QA Group stated: "we would not recommend any specific pre-trial or on-trial QA beyond the independent audit check and collection of data on the CRF."

Details of the radiotherapy technique for the four patients in the immediate radiotherapy arm who were excluded from the per-protocol analysis and developed a PTM

In 3/4 cases, the radiotherapy field size was smaller than that stipulated in the protocol (instead of a minimum field size of 7x7cm, the field sizes were: 6x8cm, 6x6cm and 6x7cm). Two of them were treated with electrons and one was treated with kv photons. All had 21Gy in 3 fractions delivered within 42 days of their pleural intervention.

One patient was randomised 36 days after their pleural intervention (ie >35 days post procedure, hence the protocol violation). However the first fraction of radiotherapy was given within the 42 day window. They received 21Gy in 3#s which was delivered using electrons. The field size was 8x8cm.

Patient experience questionnaire: Post radiotherapy questionnaire

	Immediate radiotherapy group (completed by 93/102 patients)					Deferred radiotherapy group (completed by 5/9 patients who received RT for a PTM)					
	**Not at all (n)**	**A Little (n)**	**Quite a bit (n)**	**Very much (n)**	**Mean score (SD)***	**Not at all (n)**	**A Little (n)**	**Quite a bit (n)**	**Very much (n)**	**Missing data**	**Mean score (SD)***
Did you find attending radiotherapy inconvenient?	67	21	4	1	1·34 (0·62)	3	1	1	0	0	1.6 (0.89)
Did you find attending radiotherapy reassuring?	8	21	38	25	2·87 (0·92)	1	2	1	1	0	2.4 (1.14)
Did attending radiotherapy interfere with your usual activities?	66	22	4	1	1·35 (0·62)	2	2	0	1	0	2 (1.22)
Has your quality of life been affected by radiotherapy?	66	16	9	2	1·43 (0·76)	2	0	2	0	1	2 (1.15)
Since the radiotherapy, have you felt more tired than usual?	40	35	15	3	1·80 (0·83)	2	0	1	2	0	2.6 (1.52)
Since radiotherapy, have you felt more sick than usual?	74	15	4	0	1·25 (0·52)	4	1	0	0	0	1.2 (0.45)
Since radiotherapy have you noticed any new skin changes at the radiotherapy site?	47	35	6	4	1·64 (0·79)	2	3	0	0	0	1.6 (0.55)
Since the radiotherapy, have you noticed more chest pain or discomfort than usual?	56	28	6	2	1·5 (0·72)	2	2	1	0	0	1.8 (0.84)
Any other comments	• Breathing feels easier<br>• Breathless<br>• Excessive burning but now better<br>• Felt ill for 7 days after radiotherapy<br>• Fine, no complaints<br>• First class<br>• I didn’t mind the inconvenience because it was a good cause – ie the trial<br>• I was tired for a week or two; just slight redness<br>• Itching around site<br>• Long wait on day 3<br>• No discomfort at all<br>• Nipple area became very sore and tender<br>• Pleasant reassuring staff – a great help<br>• Pleased I had it done<br>• Shortage of breath<br>• Site of treatment already feels improved<br>• Staff were first class, they were happy to accommodate time and delays. Cheerful staff, made me feel at ease. Always on time.<br>• The shorter sessions easier to cope with<br>• Treatment was very efficient<br>• Uncomfortable lying flat for a long period of time<br>• Very impressed with cancer site. Slight itchiness now gone.<br>• Very satisfied<br>• Worst feature was delayed due to appointments made when it was known that LLAB was unavailable for treatment. Reassuring in the sense that I have not been abandoned to my fate and am likely to survive a bit longer. Most of my time is spent as a carer. What quality? Tiredness may have been due to a lot of house clearance sorting auctions for collection. Arranged before radiotherapy. Possibly a slight infection picked up at hospital as I have had cold sores on my lip.<br>• No comments, just hope it works.					• Itching around site					

* Mean score generated by assuming following scores: Not at all=1; a little=2; quite a bit=3; very much=4.

Patient experience questionnaire: chest wall lump questionnaire (completed by patients who developed a PTM during trial follow up)

	**Immediate RT group (completed by 6 of 9 patients who developed a PTM)**					**Deferred RT group (completed by 13 of 16 patients who developed a PTM)**					**p value**
	**Not at all**	**A Little**	**Quite a bit**	**Very much**	**Mean score (SD)***	**Not at all**	**A Little**	**Quite a bit**	**Very much**	**Mean score (SD) ***	
In the past week, has the chest wall lump caused you pain or discomfort?	0	2	3	1	2·83 (0·75)	2	7	2	2	2·31 (0·95)	0·46
In the past week, have you felt anxious about the chest wall lump?	0	2	2	2	3 (0·89)	2	7	2	2	2·31 (0·95)	0·55
In the past week, has the chest wall lump interfered with your usual activities?	3	2	0	1	1·83 (1·17)	9	1	2	1	1·62 (1·04)	0·35
In the past week, has the chest wall lump affected your quality of life?	1	4	0	1	2·17 (0·98)	8	2	2	1	1·69 (1·03)	0·11
In the past week, have you found the chest wall lump a nuisance?	2	3	0	1	2 (1·10)	6	5	1	1	1·77 (0·93)	1·00
Any other comments	• Most discomfort in the night<br>• Feeling tight<br>• Painful while draining<br>• Sore and tight fluid drain average 500ml.					• Very itchy and quite tender to touch when pressed<br>• I think it's getting bigger<br>• Not painful unless I lie on it					
* Mean score generated by assuming following scores: Not at all=1; a little=2; quite a bit=3; very much=4.											
